# Supplementary material for: Active site specificity profiling datasets of matrix metalloproteinases (MMPs) 1, 2, 3, 7, 8, 9, 12, 13 and 14
Source: Data Brief. 2016 Feb 22;7:299–310. doi: 10.1016/j.dib.2016.02.036 (PMC4777984; doi:10.1016/j.dib.2016.02.036)
Supplement: Supplementary file 10 — Supplementary material [file mmc10.zip › WebPICS_hMMP1_T_1%/subsite_coop.html]

 

PICS results


- # About
- # Manual
- # Analysis
- # Results

- Redraw
- Seqlogo
- Dependency
- Coop

**Potential subsite cooperativity analysis for hMMP1\_T\_1%**   
Minimum difference for subsite dependency was set to +- 10 percentage-points  
Subsite dependency was checked for all positional occurences above 1 x natural abundance  
**Subsite dependency check is restricted to P3 - P3'**

|  |  |  |  |
| --- | --- | --- | --- |
| Fixed residue | Affected residue(s) | Change (percentage-points) | Vice-Versa change |
| P3\_A | P3prime\_E | 10.1 | 13.4 |
| P3\_C | P2\_L | 36.6 | 11.9 |
| P3\_C | P3prime\_S | 33.4 | 17.4 |
| P2\_A | P2prime\_Q | 12.0 | 18.4 |
| P2\_A | P3prime\_V | 18.2 | 19.0 |
| P2\_C | P1\_C | 16.0 | 29.3 |
| P2\_D | P2prime\_T | 14.3 | 19.1 |
| P2\_L | P3prime\_D | 18.5 | 22.1 |
| P2\_L | P3prime\_E | 12.9 | 19.9 |
| P2\_M | P1\_F | 14.9 | 10.7 |
| P2\_M | P1prime\_V | 32.1 | 14.6 |
| P2\_M | P2prime\_M | 14.9 | 10.7 |
| P2\_Q | P2prime\_I | 12.7 | 14.4 |
| P2\_S | P1\_G | 11.0 | 11.0 |
| P2\_S | P1prime\_M | 11.7 | 12.7 |
| P2\_W | P2prime\_H | 62.0 | 14.3 |
| P1\_A | P2prime\_A | 13.0 | 25.9 |
| P1\_A | P2prime\_L | 10.1 | 11.4 |
| P1\_D | P3prime\_H | 13.5 | 26.8 |
| P1\_G | P3prime\_V | 12.1 | 13.7 |
| P1\_H | P1prime\_V | 29.6 | 10.7 |
| P1\_N | P2prime\_M | 11.0 | 24.5 |
| P1\_N | P3prime\_V | 11.5 | 16.1 |
| P1\_Q | P2prime\_H | 14.1 | 17.3 |
| P1prime\_C | P3prime\_T | 43.9 | 15.4 |
| P2prime\_L | P3prime\_D | 13.8 | 19.6 |
| P2prime\_Q | P3prime\_H | 16.8 | 27.9 |
| P2prime\_T | P3prime\_S | 13.9 | 10.9 |
| P2prime\_V | P3prime\_T | 14.3 | 41.1 |

  
**PICS analysis of protease: hMMP1\_T\_1%**  
277 cleavage sites analyzed  
PICS library made with (T)rypsin, (G)luC or (C)hymotrypsin:   
Cutoff for graphic display: 2 x natural abundance  

|  |  |
| --- | --- |
| Positional occurences  (table for total and relative (in %) values) | Occurences relative to natural abundance (table) |
|  |  |
